# Impressions of Inlay Cavities

**Published:** 1914-07-15

**Authors:** E. B. Spalding


### IMPRESSIONS OF INLAY CAVITIES.

BY E. B. SPALDING, D.D.S.

I believe the impression and model method is going to do much to reawaken interest in the porcelain inlay and will also increase the use of the gold inlay rather than make it more complicated, as many suppose.

It has been my privilege to interest a number of men in the impression and model method for inlays and crowns and they have all said that it has improved the quality of their work and they are continually finding more uses for it.

For nearly all impressions Dr. Van Woert uses a tray especially made for each case which carries the modeling compound to place in the cavity.

I have not been able to get as satisfactory results by this method for approximal cavities as by using a specially-made matrix for each case, placing it in position between the teeth and using rather a large cone of the modeling compound. The cone is heated so that the point and outer surface of the compound, cone end, is quite soft, but the interior is still hard, or at least very resistant, and which makes a piston to force the soft portion into the cavity surrounded by the matrix. In this manner I feel that I can get greater pressure in condensing the compound into and against the walls of the cavity, at the same time covering a considerable part of the tooth outside the cavity. The pressure should be decided and firm and be held steadily for probably thirty seconds when the fingers can be released and the whole mass chilled with cold water. Where there is a mass of the modeling compound it takes a little longer to thoroughly chill than when a tray is used with a small amount of the compound.

I have rarely found a case where the air was not completely expelled from the cavity, if the compound was soft enough and considerable pressure was used. The removal of the impression usually takes some force and sometimes it is necessary to trim away excess of compound to allow of removal.

The fitting of the matrix should be carefully done so that it slips beyond the gingival cavity margin or the modeling compound will not go over the margin. It matters not how far under the gum the cavity is if the matrix goes over the margin the compound will follow it. I am speaking of approximal cavities in bicuspids and molars.

For the matrix I use a strip of platinoid 36 gauge, probably a third wider than the cavity. One end is rounded and slipped uprightly between the teeth and over the gingival edge of the cavity and under the gum if the cavity is below it. Then the free portion of the metal is bent back over the adjoining tooth. This bending over the neighboring tooth is to prevent crowding the matrix further into the gum when the modeling compound is forced to place and thereby causing discomfort to the patient, and injury to the peridental membrance.

The portion of the metal then extending out from between the teeth is bent towards the tooth to be impressed, forming a box to confine the compound. The metal is removed from the mouth, of course, to do the bending. The matrix should be of material thin enough so it will spring away from the gingival portion of the cavity and allow the compound to pass over and beyond the cavity margin. When the impression is removed the matrix may be separated from the impression but, if necessary, it can be replaced again on the impression and caught by melting a little of the compound with a hot burnisher at any convenient point.

A class of cavities of which at first it would seem very easy to obtain a good impression, viz: the labio-cervical when one margin is under the gum, was probably the hardest for me to master. Of course, as Dr. Van Woert suggests, they may be packed with gutta percha for a time when the gum will be crowded back, making the procedure simple, but that requires an extra visit for the patient which I now find unnecessary.

If a blunt cone of compound is forced into the cervical cavity the gum, instead of being forced away from the margin of the cavity, is usually bent over on to the margin and into the cavity and the margin not exposed to the modeling compound will not be outlined in the impression. By using a cone of compound as fine as a lead pencil point and softening quickly for about a quarter of an inch from the end, if inserted quickly to the center of the cavity, will strike the floor of the cavity and spread in all directions towards the walls, running under the over-hanging gum and beyond the edge of the cavity. After the first thrust of this fine point into the cavity the bulk of the cone may be firmly pressed against the gum with no fear of it not being displaced from the cavity as well as the margin.

After an impression has been secured it is wise to drop it immediately into a small glass of cool water so that if it stands in the laboratory before being packed it will not be exposed to any undue heat, as an overly-warm room or a draught from a bunsen burner which would likely change its form or dull its sharp lines.

The impression should not be removed from the water and invested until it can be packed with amalgam as soon as the plaster is hard enough to permit. For the die I am using True Dentalloy.

Care should be taken to invest the impression so that it is thoroughly supported or embedded in the plaster. If there happens to be a small crevice between the impression and the plaster, when the impression is packed with the amalgam the free mercury will be tapped out into the crevice and remain there until the model is taken out the next day, when the mercury is liable to attach itself to the model and soften it. For point of contact and bite I have given up the use of wax as too indefinite and am using modeling compound instead.

If any one is prejudiced against the indirect method, but will try it for a time, I believe he will learn enough more about cavity preparation to repay him for his trouble, and I am quite sure he will also be a convert to impression and models.

Proper cavity formation is a matter for considerable study, and there is no easier and more convincing way to study the subject than from the model of each cavity we prepare. I know that when I began the impression method, nearly four years ago, I was astonished at the imperfect form of some of my cavities after seeing the models. I thought while preparing them in the mouth I was making fine mechanical forms for retention, but the models showed the form most faulty. In several instances, when the patient came for the setting of the inlay, I re-prepared the cavity and took a new impression.

The well trained laboratory assistant is becoming a necessity to the busy dentist every day as he learns her possibilities. I do not refer to the ordinary office girl, nor to the mechanical laboratory man, who has learned his trade in the commercial dental laboratory, but to he assistant, usually a young woman, who has been carefully trained by the dentist to do, nea ly, if not all, his mechanical work according to his ideas. In much ot her work she becomes more expert than the dentist himself. If a dentist becomes proficient in all the delicate operative procedures that are expected of him he must be relieved of much, if not all, the detail of constructive work outside the mouth. If he is not relieved of it his ability as an operator is very much handicapped.

After a correct impression of a cavity is obtained the making of the model and the construction of the inlay, either for gold or porcelain, may be done by a competent trained assistant, the work being submitted to the approval of the operator from time to time if he wishes.—*Dental Summary.*—

